# Contrast-Induced Acute Kidney Injury and Endothelial Dysfunction: The Role of Vascular and Biochemical Parameters

**DOI:** 10.3390/jpm13040701

**Published:** 2023-04-21

**Authors:** Adolfo Marco Perrotta, Antonietta Gigante, Silverio Rotondi, Paolo Menè, Adriano Notturni, Stefano Schiavetto, Gaetano Tanzilli, Chiara Pellicano, Giuseppe Guaglianone, Francesca Tinti, Paolo Palange, Sandro Mazzaferro, Rosario Cianci, Silvia Lai

**Affiliations:** 1 Nephrology Unit, Department of Translational and Precision Medicine, Sapienza University of Rome, 00185 Rome, Italy; 2 Department of Translational and Precision Medicine, Sapienza University of Rome, 00185 Rome, Italy; 3 Department of Clinical and Molecular Medicine, Sapienza University of Rome, 00185 Rome, Italy; 4 Department of Public Health and Infectious Diseases, Sapienza University of Rome, 00189 Rome, Italy; 5 Department of Internal Medicine, Anesthesiology and Cardiovascular Sciences, Sapienza University of Rome, 00185 Rome, Italy; 6 Department of Chemistry and Drug Technologies, School of Hospital Pharmacy, Sapienza University of Rome, 00185 Rome, Italy

**Keywords:** contrast-induced acute kidney injury, renal resistivity index, intima-media thickness, C reactive protein, percutaneous coronary interventions, serum uric acid

## Abstract

Introduction: Contrast-induced acute kidney injury (CIAKI) is one of the main causes of acute renal failure in hospitalized patients, following the administration of iodinated contrast medium used for CT scans and angiographic procedures. CIAKI determines a high cardiovascular risk and appears to be one of the most feared complications of coronary angiography, causing a notable worsening of the prognosis with high morbidity and mortality. Aim: To evaluate a possible association between the renal resistive index (RRI) and the development of CIAKI, as well as an association with the main subclinical markers of atherosclerosis and the main cardiovascular risk factors. Materials and Methods: We enrolled 101 patients with an indication for coronary angiography. Patients underwent an assessment of renal function (serum nitrogen and basal creatinine, 48 and 72 h after administration of contrast medium), inflammation (C reactive protein (CRP), serum calcium and phosphorus, intact parathormone (iPTH), 25-hydroxyvitaminD (25-OH-VitD), serum uric acid (SUA), total cholesterol, serum triglycerides, serum glucose and insulin). All patients also carried out an evaluation of RRI, intima-media thickness (IMT), interventricular septum (IVS) and the ankle-brachial index (ABI). Results: 101 patients (68 male), with a mean age of 73.0 ± 15.0 years, were enrolled for the study; 35 are affected by type 2 diabetes mellitus. A total of 19 cases of CIAKI were reported (19%), while among diabetic patients we reported an incidence of 23% (8 patients). In our study, patients with CIAKI had significantly higher RRI (*p* < 0.001) and IMT (*p* < 0.001) with respect to the patients who did not develop CIAKI. Furthermore, patients with CIAKI had significantly higher CRP (*p* < 0.001) and SUA (*p* < 0.006). Conclusions: We showed a significant difference in RRI, IMT, SUA and CRP values between the population developing CIAKI and patients without CIAKI. This data appears relevant considering that RRI and IMT are low-cost, non-invasive and easily reproducible markers of endothelial dysfunction and atherosclerosis.

## 1. Introduction

The definition of contrast-induced acute kidney injury (CIAKI) is an absolute (≥0.5 mg/dl) or relative increase (≥25%) in serum creatinine after 48–72 h from exposure to an iodinate contrast agent [1]. The incidence differs from 1–2% [2,3,4] in patients with no risk factors, to 10–20% in patients with diabetes mellitus or chronic kidney disease (CKD) [5,6]. The average CIAKI incidence has decreased from 15 to 7% in the last ten years [2]. It is probably due to greater knowledge about associated risk factors, preventive measures and prompt diagnosis. The incidence of CIAKI depends on the presence of patient-related risk factors as well as factors from the procedure (high dose of contrast medium, intra-arterial route of administration).

Generally, CIAKI results in mild and transient acute kidney injury (AKI), with some patients requiring dialysis, which increases the mortality rate [7,8]. The main risk factors associated with CIAKI are pre-existing kidney failure, multiple myeloma, CKD, diabetes, atheroembolic disease, heart failure, myocardial infarction, hypovolemia, older age, anemia, peripheral vasculopathy, arterial hypertension and use of the counter-pulsator [9,10].

In the renal parenchyma, iodinated contrast medium seems to determine transient vasoconstriction of the afferent, efferent and vasa arterioles recta; the hypoxic status could result in tubular damage [11]. Another pathogenetic mechanism could be the direct toxic effect against tubular cells, which leads to cellular apoptosis [12]. In consideration of the important role played by renal vasoconstriction in the pathogenesis of CIAKI, we found it interesting to evaluate a pre-existing increased resistance to the renal arterioles that could prove to be a condition favoring the development of this pathology, through the evaluation of an index of renal vascularization (renal resistive index (RRI)). Furthermore, considering the higher cardiovascular risk of patients who develop CIAKI, it may be important to evaluate other cardiovascular indices such as intima-media thickness (IMT).

### Aim of the Study

The aim of the study was to evaluate the association between RRI and the onset of CIAKI. We also evaluated subclinical markers of atherosclerosis (IMT, interventricular septum (IVS), the ankle-brachial index (ABI)), the main cardiovascular risk factors such as C reactive protein (CRP), mineral metabolism (serum calcium and phosphorus, intact parathormone (iPTH), 25-hydroxyvitaminD (25-OH-VitD)) and metabolic indexes (serum uric acid (SUA), total cholesterol, serum triglycerides, serum glucose and insulin) as possible predictive factors of CIAKI.

## 2. Materials and Methods

The study was conducted in accordance with the Declaration of Helsinki and approved by the Institutional Review Board Sapienza University of Rome, Italy (Approval code: 6.1 2018).

The study conforms to the principles outlined in the Declaration of Helsinki and we obtained written informed consent. We performed a longitudinal, observational study on 101 hospitalized patients at the University Hospital “Policlinico Umberto I” of Rome (Sapienza University of Rome, Italy). We enrolled consecutive patients of both sexes, aged ≥ 18 years, with an estimated glomerular filtration rate (eGFR) between 45 mL/min and 90 mL/min (CKD-EPI) admitted in the Cardiology Dept., Angiology Dept. and Intensive Care Unit candidate to coronary angiography. For CIAKI prevention, according to protocol, saline solution (500 mL) was administered to all patients before the procedure. Metformin was discontinued 48 h before the exam; angiotensin-converting enzyme (ACE) inhibitor/angiotensin receptor blocker (ARB) and diuretics were discontinued 24 h before the exam. We excluded patients with ongoing sepsis, current infections, hemodynamic instability, hepatorenal syndrome, stenosis and/or hemodynamic obstruction of the renal artery or who refused to consent. Before the angiographic procedure, some clinical and laboratory evaluations were performed.

### 2.1. Laboratory Measurements

In all patients, creatinine levels were evaluated (mg/dL) the day before (T0), and 48 and 72 h after administration of the contrast media. An increase of ≥0.5 mg/dl or ≥25% was considered diagnostic for CIAKI. Other laboratory tests at T0 were: fasting plasma glucose (mg/dl), serum insulin (µU/mL), serum nitrogen (mg/dl), SUA (mg/dl), serum calcium (mg/dl), serum phosphorus (mg/dl), serum sodium (mEq/L), serum potassium (mEq/L), hemoglobin (g/dl), total serum cholesterol (mg/dl), triglycerides (mg/dl), high-density lipoprotein (HDL) (mg/dl), low-density lipoprotein (LDL) (mg/dl) and CRP (mg/dl). 25-OH-VitD (ng/mL) was assessed using radioimmunoassay and iPTH (pg/mL) using III generation assay. The eGFR was calculated with the abbreviated chronic kidney disease epidemiology formula (CKD-EPI), as defined by Levey et al. [13]. Arterial blood gas was performed using a blood gas analyzer (Nova Phox Plus C, Prospect Street, Waltham, MA, USA).

### 2.2. Blood Pressure Measurements

Clinic blood pressure (BP) measurements were performed three times after 10 min of rest in a seated position using a standard automated sphygmomanometer and cuffs adapted to the arm’s circumference, as per 2017 Cardiology/American Heart Association (ACC/AHA) Hypertension Guidelines. Hypertension was defined by an average systolic BP (SBP) ≥ 130 mmHg or diastolic BP (DBP) ≥ 80 mmHg, on repeated measurements. We considered for statistical analysis the BP values detected before the procedure [14].

### 2.3. Carotid Intima-Media Thickness Assessment

Carotid artery imaging was performed before the procedure by a single investigator with a high-resolution B-mode ultrasound machine Toshiba Aplio xV (Toshiba Aplio xV; Toshiba American Medical Systems, Inc., Tustin, CA, USA) equipped with a 5- to 12-MHz linear transducer with a 0.01 mm resolution, following a standardized vascular protocol. IMT was sampled in three sites on both right and left sides: internal carotid artery (ICA), carotid bulb and common carotid artery (CCA). Images were captured in end-diastole triggered by electrocardiographic recording. The mean value was calculated and considered normal between 0.55 and 0.9 mm [15].

### 2.4. Renal Resistive Index (RRI)

Participants were studied with the high-resolution B-mode ultrasound machine Toshiba Aplio xV (Toshiba Aplio xV, Toshiba American Medical Systems, Inc., Tustin, CA, USA) equipped with a 3–3.5 MHz convex transducer. RRI mean values were assessed with three different measurements in the renal superior pole, interpolar regional and inferior pole on interlobular and interlobar or arcuate arteries in both kidneys. We used an anterior and oblique approach to detect the renal arteries and intra-parenchymal vessels. Three to five reproducible and consecutive waveforms with similar aspects from each kidney were obtained [16,17]. We determined the peak systolic velocity and end-diastolic velocity (centimeters/second) to calculate the RRI as = [1 − (end-diastolic velocity/maximal systolic velocity)] × 100. The intra-reader correlation coefficient for RRI was 0.97, whereas the inter-reader was 0.92.

### 2.5. Echocardiography

All patients underwent echocardiographic examination to assess the morphology and performance of the heart. We used a Philips model iE33 xMATRIX equipped with the S5-1 transducer. We evaluated the IVS thickness as well as the posterior wall thickness at end-diastole (normal range for male 0.6–1 cm and for female 0.6–0.9 cm) and the end-diastolic left ventricular (LV) dimension in M-mode and in parasternal long axis (normal range 42–58.4 mm for male and 37.8–52.2 mm for female). The following parameters were assessed: LV mass index and the relative wall thickness, the presence of concentric or eccentric hypertrophy and the presence of diastolic dysfunction. In addition, we evaluated the end-systolic and diastolic volume (63–150 mL and 21–61 mL for males, 46–106 mL and 14–42 mL for females), using Simpson’s biplane method to estimate the ejection fraction (normal range 52–72% for male and 54–74% for female) [18].

### 2.6. Statistical Analysis

Statistical analysis was performed using SPSS 26 software (Bioz, Los Altos, CA, USA). The Shapiro–Wilk test was used to evaluate the normal distribution of data. Continuous variables were expressed as mean ± standard deviation (SD). Categorical variables were expressed as absolute frequency and percentage. Differences between groups were evaluated by Student’s or Mann–Whitney’s *t*-test. Differences between categorical variables were evaluated by the chi-square or Fisher’s exact test. Stepwise logistic regression analysis was used to evaluate the association between a dependent dichotomic variable (CIAKI yes or no) and continuous independent variables (age, IMT, RRI, eGFR, creatinine, SIV, CRP, SUA) or categorical independent variables (gender). Results were expressed as odds ratio (OR) and 95% confidence interval (95% CI). A *p*-value < 0.05 was considered significant.

## 3. Results

A total of 101 consecutive patients (68 male), with a mean age of 73.0 ± 15.0 years, were enrolled on the study. The mean eGFR at baseline was 69.50 (±22.62) and 35 patients (34.6%) were affected by diabetes mellitus. Patients had a mean RRI of 0.68 (±0.05), mean ABI of 1.06 (±0.11) and mean IVS of 11.71 mm (±1.83). Table 1 shows the demographic and clinical characteristics of patients enrolled in this study.

In Table 2 is shown the comparative analysis of demographic and clinical characteristics of patients with (CIAKI+) and without (CIAKI−) CIAKI. A total of 19 cases of CIAKI were reported (19%); eight of them were affected by diabetes mellitus, with an incidence of 23% in the group of diabetic patients. Patients with diabetes mellitus were evenly distributed between CIAKI+ and CIAKI—(23% vs. 32.9%, *p* = 0.345).

The group CIAKI+ had significantly higher RRI (*p* < 0.001), IMT (*p* < 0.001), CRP (*p* < 0.001) and SUA (*p* < 0.006). We did not find any significant differences in ABI (*p* = 0.150), IVS (*p* = 0.257), total cholesterol (*p* = 0.158), serum glucose and insulin (*p* = 0.777 and *p* = 0.307, respectively).

As at the univariate analysis there was not any independent risk factor for the onset of CIAKI, we did not perform multivariate analysis (Table 3).

## 4. Discussion

In our study, the incidence of CIAKI was 19%, which is in accordance with the literature [2,6]. CIAKI is accompanied by high in-hospital mortality, about 34% compared to 7% among patients who did not develop it [19]. Rihal et al. also showed that one-year and five-year mortality would be three times higher in patients with CIAKI occurrence [20]. Many factors have been found to be involved in the pathogenesis of CIAKI. The intra-arterial iodine contrast causes transient vasodilation due to the release of nitric oxide from the endothelium [12]. Then, arteriolar vasoconstriction occurs for several seconds or minutes, at the level of the peripheral circulation [11], but at the renal level, this sustained vasoconstriction can last for a few hours, at the level of the afferent and efferent arteriole and of the vasa recta [12]. The patients most at risk of developing CIAKI are mainly those with CKD and diabetes; in fact, when a reduced functional reserve of nephrons is present, the reduction in blood flow secondary to vasoconstriction after administration of the contrast medium, can compromise the oxygenation of the external medulla of the kidney, with consequent ischemia in the proximal and distal tubules [12]. Additionally, in our study, 23% of diabetic patients enrolled developed CIAKI. A second pathogenetic mechanism can be a direct cause of damage to the contrast medium, of the apical surface of the proximal tubular cells and of the basolateral surface in the interstitial tubule space which can lead to balloon-like degeneration and subsequently apoptosis. The mitochondria of damaged renal tubular cells release unbound iron, intracellularly, which can produce free radicals and can promote oxidative stress [11]. Considering the direct toxic effects on vascular cells by iodinate contrast medium, we considered RRI to evaluate the kidney vascularization status in patients who develop CIAKI-. In our cohort of patients, we observed higher mean RRI, IMT, CRP and uric acid in CIAKI+ compared to CIAKI−. However, these factors did not predict onset of CIAKI in our cohort, probably due to a small sample size, although in the literature there is contrasting evidence on the role of RRI as a predictive factor for CIAKI [21,22]. Additionally, impairment of intrarenal hemodynamic parameters evaluated by RRI is often associated with composite outcome, with a combination of high RRI and eGFR during follow-up. The absence of composite outcomes during follow-up could be another explanation for the lack of predictor factors of CIAKI in the present study. Different results are probably due to extrarenal factors able to influence RRI. Therefore, RRI should not be considered exclusively as a marker of kidney damage, moreover a cardiovascular outcome parameter [23]. We also reported a significant increase in IMT in patients CIAKI+. IMT is a marker of subclinical atherosclerosis. In addition, RRI and IMT are indicators of endothelial dysfunction, an important mechanism involved in CIAKI pathogenesis [11,12,24,25]. In our study, we also reported a significant difference in CRP and SUA between the two groups. Both are related to the development of endothelial dysfunction, enhancing contrast medium toxicity. In particular, CRP can directly modify vascular endothelium-inducing adhesion molecules, reducing nitric oxide (NO) in endothelial cells and affecting LDL metabolism [26]. For these reasons, CRP level is an independent risk factor for cardiovascular events [27]. SUA could play a role in oxidative stress, leading to endothelial dysfunction and promoting the development of inflammation and atherosclerosis [28,29].

We conducted an observational study in a small cohort of patients. Considering the relevant impact of CIAKI on hospitalization length, morbidity and mortality, it is necessary to set larger and multicentric studies.

## 5. Conclusions

Our study showed higher RRI, IMT, CRP and uric acid in CIAKI+ compared to CIAKI- patients.

Additional studies are necessary to confirm these results in larger series of patients.

## Figures and Tables

**Table 1:** Patients’ characteristics. Data are shown as mean ± standard deviation or number (%).

Age (Years)	73 ± 15
F/M	33 (32.7)/68 (67.3)
eGFR (ml/min/1.73 m^2^)	69.50 ± 22.62
IMT (mm)	0.09 ± 0.01
RRI	0.68 ± 0.05
ABI	1.06 ± 0.11
IVS (mm)	11.71 ± 1.83
Diabetes mellitus	35 (34.6)
Hemoglobin (g/dl)	12.93 ± 1.75
Serum Uric Acid (mg/dl)	7.22 ± 4.84
Total Cholesterol (mg/dl)	154.02 ± 38.25
iPTH (pg/mL)	61.04 ± 41.93
25-OH-VitD (ng/mL)	17.44 ± 11.98

Abbreviations: eGFR, estimated Glomerular Filtration Rate; IMT, Intima Media Thickness; RRI, Renal Resistive Index; ABI, Ankle Brachial Index; IVS, interventricular septum; iPTH, intact Parathormone; 25-OH-VitD, 25-hydroxyvitaminD.

**Table 2:** Comparative analysis of demographic and clinical characteristics of patients with and without CIAKI. Data are shown as mean ± standard deviation or number (%). In bold are reported significant results.

	CIAKI+ (*n* = 19)	CIAKI− (*n* = 82)	*p* Value
Age (years)	69 ± 10	73 ± 11	0.149
eGFR (ml/min/1.73 m^2^)	80.7 ± 24.0	70.1 ± 22.8	0.07
IMT (mm)	0.97 ± 0.3	0.6 ± 0.16	**<0.001**
RRI	0.78 ± 0.10	0.67 ± 0.05	**<0.001**
ABI	1.03 ± 0.14	1.07 ± 0.10	0.150
IVS (mm)	11.3 ± 2.2	11.8 ± 1.6	0.257
Systolic blood pressure (mmHg)	130 ± 6	131 ± 5	0.876
Diastolic blood pressure (mmHg)	80 ± 4	79 ± 3	0.789
EF (%)	54 ± 4	53 ± 6	0.867
Hemoglobin (g/dl)	13.2 ± 1.5	12.6 ± 1.7	0.202
CRP (mg/L)	4 ± 3.4	0.8 ± 1.4	**<0.001**
Serum Uric Acid (mg/dl)	10.8 ± 7.6	6.9 ± 4.9	**<0.01**
Total Cholesterol (mg/dl)	143.0 ± 32.4	157.0 ± 40.0	0.158
Serum glucose (mg/dl)	109.0 ± 30.0	112.2 ± 47.0	0.777
Serum Insulin (µU/mL)	12.0 ± 6.7	15.0 ± 12.3	0.307
iPTH (pg/mL)	66.0 ± 31.0	63.0 ± 45.0	0.783
25-OH-VitD (ng/mL)	20.5 ± 16.6	16.9 ± 13.0	0.305

Abbreviations: CIAKI− = patients not developed contrast-induced acute kidney injury; CIAKI+ = patients developed contrast-induced nephropathy; eGFR, estimated Glomerular Filtration Rate; IMT, Intima Media Thickness; RRI, Renal Resistive Index; ABI, Ankle Brachial Index; IVS, interventricular septum; EF, ejection fraction; CRP, C reactive protein; iPTH, intact Parathormone; 25-OH-VitD, 25-hydroxyvitaminD. Significant *p* values are reported in bold.

**Table 3:** Stepwise logistic regression analysis with odds ratio (OR) and 95% confidence interval (CI).

	OR (CI)	*p*
Age	0.969 (0.935–1.004)	>0.05
Gender	1.085 (0.458–2.566)	>0.05
IMT	0 (0–30677867)	>0.05
RRI	0.024 (0–35.939)	>0.05
eGFR	1.015 (0.996–1.034)	>0.05
creatinine	1.039 (0.536–2.014)	>0.05
IVS	0.839 (0.654–1.076)	>0.05
CRP	1 (1–1)	>0.05
Serum Uric Acid	1.054 (0.887–1.253)	>0.05

Abbreviations: eGFR, estimated Glomerular Filtration Rate; IMT, Intima Media Thickness; RRI, Renal Resistive Index; IVS, interventricular septum; CRP, C reactive protein.

## Data Availability

The data presented in this study are available on request from the corresponding author upon reasonable request.
